# Association Between Race/Ethnicity and Survival in Women With Advanced Ovarian Cancer

**DOI:** 10.7759/cureus.16070

**Published:** 2021-06-30

**Authors:** Justin J Cheng, Bu Jung Kim, Catherine Kim, Pura Rodriguez de la Vega, Marcia Varella, Carolyn D Runowicz, Juan Ruiz-Pelaez

**Affiliations:** 1 Department of Translational Medicine, Florida International University, Herbert Wertheim College of Medicine, Miami, USA; 2 Department of Translational Medicine, Florida International University Herbert Wertheim College of Medicine, Miami, USA; 3 Department of Academic Affairs, Florida International University, Herbert Wertheim College of Medicine, Miami, USA; 4 KMC senior researcher, Kangaroo Foundation, Bogota, COL

**Keywords:** race, ethnicity, ovarian cancer, survival, health disparity

## Abstract

Introduction

Ovarian cancer is the fifth-leading cause of cancer-related mortality in US women. There are survival disparities between non-Hispanic black (NHB) and non-Hispanic white (NHW) women. We assessed if insurance status or extent of disease modified the effect of race/ethnicity on survival for ovarian cancer.

Methods

A historical cohort was assembled using the 2007-2015 National Cancer Institute’s Surveillance, Epidemiology, and End Result (SEER) dataset. Adult NHB and NHW (>18 years) diagnosed with regional and distant ovarian cancer were included. The outcome was five-year cause-specific mortality. Multivariable Cox regression models were fitted, including race by the extent of disease and race by insurance status interaction terms.

Results

For each significant interaction, separate Cox models were fitted. In total 8,043 women were included. The insurance status/race interaction was not statistically significant, but the extent of disease modified the effect of race on survival. NHB survival was lower in regional disease (adjusted hazard ratio (HR) =1.6; 95% confidence interval (CI) 1.1-2.4), while there was no difference in survival between women with distant disease (adjusted HR =1.0; 95%CI 0.9-1.2).

Conclusions

Ovarian cancer mortality is similar between NHB and NHW women with the distant disease but higher in NHB women with regional disease. Further research should clarify whether this difference is due to access to quality cancer treatment or other factors affecting treatment response.

## Introduction

In 2018 ovarian cancer accounted for 1.3% of all new cancer cases in the United States (US) with epithelial subtype representing 90% of all ovarian cancers [1]. Despite its relatively low incidence, ovarian cancer is the fifth-leading cause of cancer-related death in US women. Given its insidious onset, ovarian cancer is often detected at stage III or IV disease, with over half of women diagnosed when the cancer has metastasized. The 5-year relative survival among women diagnosed with ovarian cancer from 2011 to 2017 was 49%. For those with localized disease it was 93%, while for those with regional ovarian cancer (cancer spread limited to pelvic and regional lymph nodes) it was 75%, and for those others with distant metastases it was only 30% [2].

Despite marked improvements in ovarian cancer treatment, there continues to be a survival difference between non-Hispanic black (NHB) and non-Hispanic white (NHW) women. From 2000 to 2018, the 5-year relative survival in NHB women (22%) was lower than that of NHW women (29%) [3, 4]. Proposed reasons for the disparity in mortality include reproductive and biological variability, socioeconomic factors, racial/ethnic differences, and access to quality cancer care and treatment [5-10].

Studies consistently report lower survival among NHB women compared with NHW women [11-13] and significantly increased mortality in NHB compared to other ethnic groups after controlling for socioeconomic and other prognostic factors [14]. One systematic review found worse survival in NHB compared to NHW women after 1985, when platinum-based chemotherapy and surgical debulking was accepted as standard practice [15]. A single-institution study that adjusted for stage, optimal surgical debulking, and platinum sensitivity found no difference in survival following primary treatment between NHB and NHW women [16]. After receiving quality care, including surgery and chemotherapy, NHB and NHW women did not differ in survival in this study. This suggests that access to quality cancer care is likely partly responsible for the race specific survival differences. Previous population-based studies have found that lack of insurance is associated with reduced likelihood of receiving local treatment [17, 18]. Additionally, one study found a survival difference in earlier stages but not advanced stages for NHB and NHW women [19]. While previous studies have assessed potential reasons for the survival disparity, there were no known studies that assessed for both insurance status and extent of disease as modifiers of the survival disparity between NHB and NHW women.

The present study was designed to investigate whether the association between race/ethnicity with survival in NHB and NHW women with regional and distant epithelial ovarian cancer is modified by insurance status, and/or by extent of disease at diagnosis. We hypothesized that the direction and magnitude of the association between race and survival in women with epithelial ovarian cancer differs among subgroups defined by insurance status and/or extent of disease.

## Materials and methods

Study design

In this secondary data analysis, we assembled a historical cohort using data from the 2007- 2015 Surveillance, Epidemiology, and End Results (SEER) database. Briefly, SEER is a surveillance system of the National Cancer Institute (NCI) that routinely collects information and publishes cancer incidence and survival data from population-based cancer registries, covering approximately 28% of the population in the United States. For ovarian cancer, the database defines regional disease as a disease confined to the pelvis, including the regional lymph nodes, and distant disease as spread from the primary tumor to distant organs, tissues, and/or non-regional lymph nodes. Localized disease cases, defined as confined to the ovary, were excluded due to the marked difference in survival and perhaps biology [20].

Participants

The study investigated US NHW and NHB women aged 18 and over with a single primary diagnosis and classified as regional or distant epithelial ovarian cancer initially diagnosed from 2007 to 2015. Other races and ethnicities were excluded to specifically analyze disparities between NHW and NHB women. Younger women (less than age 65 years) were categorized by age groups of 18-50 and 51-64 to delineate pre- and post-menopausal women, as 51 years is the average age of menopause in the United States [21]. The SEER database did not include information on menopause. Participants with the following conditions were included: epithelial ovarian histology of invasive behavior (code C569 (International Classification Diseases for Oncology, 3rd Edition [ICD-O-3] included serous (8020, 8021, 8022, 8050, 8260, 8441, 8442, 8450, 8460, 8461, 8462, 8463, 9014); endometrioid (8380, 8381, 8382, 8383, 8482, 8383, 8482, 8570); mucinous (8470, 8471, 8472, 8480, 8481, 9015); clear cell (8310, 8313); carcinosarcoma (8575, 8950, 8951, 8980, 8981), malignant Brenner carcinoma (9000, 8010, 8140, 8230, 8440), and mixed (8255, 8323) [22]. Transitional cell carcinoma was excluded due to the low-frequency distribution of this histologic subtype leading to unnecessary heterogeneity with little power to address it.

The extent of disease was defined as regional or distant according to the SEER historic staging system rather than American Joint Committee on Cancer (AJCC) staging system, which was only available after 2010. Race/ethnicity groups other than NHW and NHB women were excluded from this study due to the low representative sample within the SEER database. The study also excluded participants who did not have information on race, staging, insurance status, survival, cases with the unknown or missing cause of death, cases with no information on survival time, and those cases in which the diagnosis of ovarian cancer was established after death (death certificate or autopsy), and therefore, had no survival time after diagnosis.

Variables

The exposure of interest in this study was race/ethnicity: non-Hispanic White (NHW) and non-Hispanic Black (NHB). The outcome was a 5-year cause-specific survival time, computed as the time from diagnosis to cause-specific death or to censoring (dying from a different cause, lost to follow up, or remaining alive after 5 years). The potential effect modifiers included insurance status (uninsured/Medicaid or insured/nonspecific insured) and extent of disease. Potential confounders that were tested and eventually controlled for included age at diagnosis, histologic type, grade, marital status, and surgery.

Grading was defined as low grade (grade 1 or 2), high grade (grade 3 or 4), or unknown grade (no information available). Marital status was defined as partnered if married or unmarried/domestic partner, and unpartnered if single, separated, divorced, or widowed. Surgery was defined as any kind of cancer-directed surgery. No specific information on the type of cancer surgery was available in the dataset. We did not adjust for hospital characteristics or volume or chemotherapy as they were not included in SEER.

Statistical methods

We profiled the distributions of the variables of interest and described the characteristics of the study population. Bivariate analysis was used to describe the distribution of variables according to exposure. Discrete control variables were compared using the Chi-square test. Patient 5-year cause-specific survival (CSS) was calculated using the Kaplan-Meier method. SEER defines cause-specific survival as a net survival measure representing the survival of a specified cause of death in the absence of other causes of death. It accounts for causes of death in conjunction with tumor sequence, site of the original cancer diagnosis, and comorbidities. Differences between survival times across groups were tested for statistical significance using the log-rank test. A two-sided p-value < 0.05 was considered statistically significant. Confounders were defined as factors that were found to be unevenly distributed between exposure groups and statistically associated with survival.

A multivariable Cox regression model was fitted to compute and compare adjusted estimations of 5-year cause-specific mortality rates between NHB and NHW, adjusting for potential confounders. Potential first-order interaction terms (race-by-insurance status and race-by-extent of disease) were included and tested for significance. If significant interactions were found, only stratified models were presented. All statistical analyses were performed using Stata® 15.0 (StataCorp). 

Ethics

The study was considered non-human subjects research and therefore exempt from Institutional Review Board (IRB) review (IRB-20-0080).

## Results

**Figure 1:**
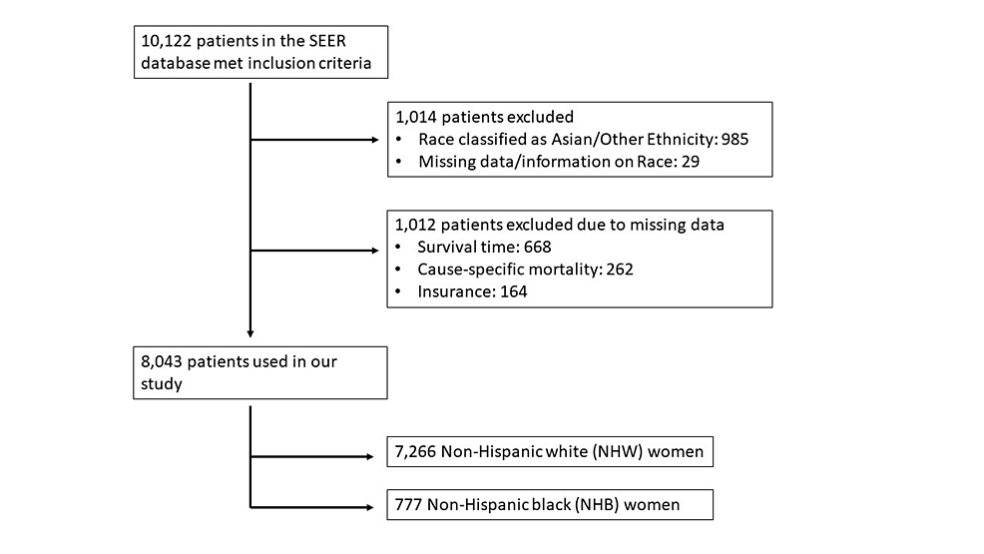
Patients with ovarian cancer who were included in the study cohort (2007-2015) SEER indicates the National Cancer Institute’s Surveillance, Epidemiology, and End Results Program

The 2007 to 2015 SEER dataset included 10,122 women over the age of 18 years diagnosed with regional or distant primary epithelial ovarian cancer. Of the 10,122 women with regional or distant epithelial ovarian cancer, 8043 met our inclusion criteria (Figure 1).

All baseline characteristics except the extent of disease (p =0.051) were significantly different between NHB and NHW women. The NHB group had a greater proportion of women age 18-50 and 51-64 years, a larger proportion of unpartnered women, and a greater proportion of uninsured/Medicaid individuals (Table 1). Furthermore, NHB women had a larger proportion of those with unreported grade and a higher proportion of malignant Brenner histologic type. A smaller proportion of NHB women received surgery for initial treatment compared to NHW women (66.8% v. 81.6%, p <0.001).

**Table 1:** Baseline characteristics of non-Hispanic white and non-Hispanic black women with regional or distant ovarian cancer (N=8,043) a – “Partnered” is defined as married or unmarried/domestic partner, while “Unpartnered” is defined as single, separated, divorced, widowed; b – “insured” is defined as insured or insured/No specifics; c –“low” grade is defined as Grade I, well differentiated, differentiated, NOS or Grade II, moderately differentiated, intermediate differentiation, “high” grade is defined as Grade III, poorly differentiated or Grade IV, undifferentiated, anaplastic, while “undefined” is defined as cell type not determined, not stated or not applicable

Table 1: Baseline characteristics of non-Hispanic white and non-Hispanic black women with regional or distant ovarian cancer (N=8,043)					
Characteristics	Non-Hispanic White (NHW)		Non-Hispanic Black (NHB)		Significance
	N (% of NHW)		N (% of NHB)		P-value
Age (years)					0.008
18 – 50	1040	(14.3)	123	(15.8)	
51 – 64	2716	(37.4)	324	(41.7)	
≥65	3510	(48.3)	330	(42.5)	
Marital status^a^					<0.001
Partnered	3976	(56.5)	233	(31.2)	
Unpartnered	3065	(43.5)	515	(68.9)	
Insurance^b^					<0.001
Uninsured/Medicaid	623	(8.6)	170	(21.9)	
Insured	6643	(91.4)	607	(78.1)	
Grade^c^					<0.001
Low	749	(10.3)	62	(8.0)	
High	4246	(58.4)	374	(48.1)	
Undefined	2271	(31.3)	341	(43.9)	
Extent of Disease					0.051
Regional	935	(12.9)	81	(10.4)	
Distant	6331	(87.1)	696	(89.6)	
Histologic Type					<0.001
Serous	4669	(64.3)	433	(55.7)	
Endometrioid	391	(5.4)	41	(5.3)	
Mucinous	149	(2.1)	25	(3.2)	
Clear cell	285	(3.9)	20	(2.6)	
Carcinosarcoma	301	(4.1)	40	(5.2)	
Malignant Brenner carcinoma	1127	(15.5)	196	(25.2)	
Mixed	344	(4.7)	22	(2.8)	
Surgery					<0.001
Yes	5928	(81.6)	518	(66.8)	
No	1334	(18.4)	257	(33.2)	

**Figure 2:**
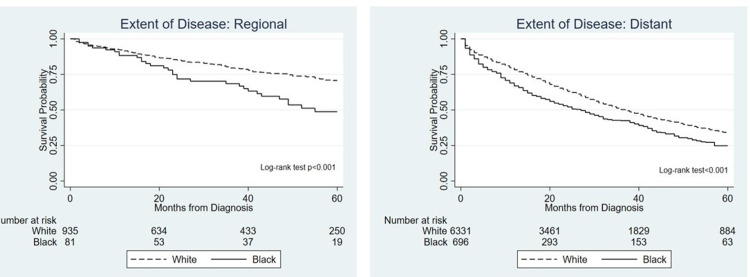
Kaplan Meier curves of survival estimates of patients with regional and distant ovarian cancer

Prior to multivariable adjustment, NHB race was associated with a 40% increase in the hazard of death as compared to NHW (hazard ratio, HR 1.4 95% confidence interval, CI 1.3-1.6), a difference that was also evident when comparing unadjusted survival functions by means of Kaplan-Meier curves (Figure 2). A multivariable model including potential confounders and first-level interaction terms showed no significant interaction between race and insurance status (p =0.792); however, there was a significant interaction between race and extent of disease (p =0.013). Because of this interaction, separate models were run for each level of extent of disease (regional and distant) and adjusted for age, marital status, grade, histologic type, and receipt of surgery.

For women with regional disease, NHB race was associated with a 90% increased death hazard (HR 1.9 95%CI 1.3-2.8), which decreased slightly but remained significant after adjustment (HR 1.6 95% CI 1.1-2.4). In contrast, for women with distant disease, and after adjusting for confounders there was no significant difference in the hazard rates (Table 2).

**Table 2:** Unadjusted and Adjusted Cox-proportional hazards models after stratification by the extent of disease for ovarian cancer-specific 5-year survival a – after adjustment to age groups, marital status, insurance status, grade, histologic type, receipt of surgery; b – hazard ratio (HR); c – confidence interval (CI)

Table 2: Unadjusted and Adjusted Cox-proportional hazards models after stratification by extent of disease for ovarian cancer specific 5-year survival		
Extent of Disease	Unadjusted	Adjusted^a^
	HR^b^ (95% CI^c^)	HR^b ^(95% CI^c^)
Regional		
Non-Hispanic White	ref	ref
Non-Hispanic Black	1.9 (1.3-2.8)	1.6 (1.1-2.4)
Distant		
Non-Hispanic White	ref	ref
Non-Hispanic Black	1.4 (1.2-1.5)	1.0 (0.9-1.2)

## Discussion

This study assessed disparity in survival between NHB and NHW women with regional or distant epithelial ovarian cancer in the SEER 2007-2015 dataset. We compared 5-year cause-specific mortality between NHB and NHW women and assessed if there was a difference in survival based on regional or distant disease. These data suggest that racial disparity in disease-specific survival exists between NHB and NHW women. Despite the similar distribution of extent of disease at diagnosis across race groups, there is a noticeable difference in survival between NHB and NHW women with regional disease; however, this difference is no longer apparent with distant disease.

Our study found a difference in survival between NHB and NHW women, consistent with studies by Wright et al [11]; Chan et al [12], Terplan et al [15], and Stewart et al [23]. This study differs from these studies in that extent of disease appears to modify the association between race/ethnicity and survival. Prior studies on ovarian cancer using the SEER database did not assess for the extent of disease as an effect modifier of the association between race and survival [9, 24, 25]. In their single-institution study, Kim et al found that NHB women with cancer limited to the ovaries and contained within the pelvic cavity experienced worse survival than NHW women, while NHB women with cancer outside the pelvis and distant metastases experienced similar survival. The present study confirmed this finding but in a national database [19].

We examined insurance status as one possible indicator of access to quality care. In our study, NHB women had lower proportions of insurance. This disparity in insurance status appeared to explain the disparity in survival between NHB and NHW ovarian cancer patients. However, when we tested insurance status as a modifier of the association between race and survival, there was no significant effect. Insurance status as a moderator may not be statistically significant, but there still may be a difference in access to quality evidence-based care. Other factors impacting access to care are likely contributing to the disparity in survival. 

There is not a definitive reason for why survival differs with the regional disease but appears similar with distant disease. In the present study, one of the covariates that was adjusted for could partially explain the unadjusted survival disparity with distant disease. Several studies have investigated tumor biology, surgical treatment, medical comorbidities, post-operative care, and treatment, or any combination of these factors [7, 15, 26-28]. Grant et al [26] explored potential biological causes to explain the disparity. However, there is an argument against biology as the main factor since no significant difference in survival was found between NHW and NHB from 1973 to 1982, a period before modern ovarian cancer treatment was widely implemented [13]. There may be a greater opportunity for successful surgical cytoreduction with regional ovarian cancer. Previous studies have demonstrated the association between the black race and failure to receive “standard of care” treatment [8, 29, 30]. In the present study, we found that NHB patients were less likely to receive surgery for initial treatment compared to NHW patients. Failure to receive quality cancer treatment at less advanced disease may be one explanation for survival disparity at regional but not distant disease. Subsequent studies have evaluated various socioeconomic factors including income level and access to care [8, 9, 23]. Social determinants of health may be more of a barrier for NHB women with regional disease than for NHB women with distant disease. More investigation is needed to assess why NHB have worse survival than NHW in regional disease but appear to have equally poor survival in distant disease.

There were several limitations of our study. Despite SEER providing a large sample, there is potential for unreported cases and preferential reporting leading to a biased representation of all ovarian cancer patients. There was a difference in frequency distribution between NHB and NHW women, which may reflect inequity in care leading to under-diagnosis in NHB women. Residual confounding remains from potential confounders not measured in the registry such as the participant’s previous medical history and details regarding the extent of surgical treatment. Moreover, since the study is dependent on provider-reported race, an imperfect assessment of race may weaken our conclusion. Another potential limitation is the use of the extent of disease instead of tumor-node-metastasis (TNM) staging. This may make clinical application and comparison to other work challenging. Nevertheless, the local-regional-distant (LRD) staging system used by SEER is an internationally accepted standard for summarizing, reporting, and comparing cancer vital statistics [2]. Lastly, an important limitation is the lack of data available in the SEER database on chemotherapy administration, hospital characteristics, hospital volume, and other critical information. The lack of chemotherapy data may explain some of the survival differences between NHB and NHW women. Patients with distant disease also have a graver prognosis and are likely less impacted by these factors. In contrast, timely treatment with sound evidence-based interventions is likely to increase survival in regional disease. 

In our study after adjustment, we found that NHB women had worse survival than NHW women with regional disease, but survival appeared to be similar between the two groups with distant disease. The disparity in survival between NHB and NHW women with regional ovarian cancer warrants further investigation. Detailed information regarding surgical and chemotherapy treatments should be collected. There should be further exploration of other effect modifiers of the association between race and survival in ovarian cancer, as they are not reported frequently in the literature. Other factors measuring access to care should be included to assess the effects of treatments based on the stage of the disease. The goal is to identify modifiable variables associated with the disparity in survival from ovarian cancer and intervene at a public health and policy level.

## Conclusions

There is a survival disparity between non-Hispanic black and non-Hispanic white women with advanced ovarian cancer. Insurance status was not a significant effect modifier of the association between race/ethnicity and survival. The extent of the disease, however, did significantly modify the association. Non-Hispanic black women have worse survival than non-Hispanic white women for regional disease; however, the survival appears to be similar between the two groups for distant disease. Access to quality cancer care and other factors may play a role in these findings.
